# 1238. Evaluating the Impact of an Automatic Antibiotic De-escalation Protocol at an Acute Care Hospital

**DOI:** 10.1093/ofid/ofad500.1078

**Published:** 2023-11-27

**Authors:** Albert Diep, Jacob Leonard, Jonathan Cho, Stephen E Chromi, Kelli Kronsberg

**Affiliations:** MountainView Hospital, Las Vegas, Nevada; Sarasota Memorial Hospital, Sarasota, Florida; Physician Affiliate Group of New York, McKinney, Texas; MountainView Hospital, Las Vegas, Nevada; MountainView Hospital, Las Vegas, Nevada

## Abstract

**Background:**

Previously, pharmacist-led stewardship programs have been shown to result in more timely de-escalation of antibiotics and lack of adverse outcomes in patients treated for pneumonia in the intensive care unit (ICU). This study evaluated the effects of an automatic pharmacist-led antimicrobial de-escalation policy outside of the ICU.

**Methods:**

This was a retrospective pre- and post-implementation study at a 425-bed tertiary care hospital in Las Vegas, Nevada. Inclusion criteria consisted of patients admitted from October 2017 to October 2021 who had *Escherichia* sp.*, Proteus* sp.*, Klebsiella* sp., *Pseudomonas* sp., or *Enterococcus* sp. isolated from specified sites that were susceptible to certain antibiotics included in the de-escalation policy. Exclusion criteria included concomitant infections, polymicrobial infections with organisms outside of those mentioned in the inclusion criteria, deep-seated infections, or hemodynamic instability. The primary outcome was time to de-escalation, which was calculated by determining elapsed time between culture susceptibility results and start of the new antibiotic if de-escalated. If de-escalation did not occur, then time to de-escalation was calculated by comparing time of susceptibility results to antibiotic discontinuation. Secondary outcomes evaluated included rates of antibiotic de-escalation, occurrence of readmission at 30- and 90-days, incidence of all-cause inpatient mortality, length of stay, incidence of *Clostridioides difficile* infection, and time to clinical resolution. Time to clinical resolution was defined by when patient met pre-specified laboratory values following the first antibiotic dose.

**Results:**

A total of 174 patients were included in the pre-group and 52 in the post-group. The results are shown in Table 1; there was a statistically significant decrease in time to de-escalation, rates of de-escalation, and 90-day readmission rates favoring the post-intervention group, with no differences seen in the other outcomes.

**Figure:**
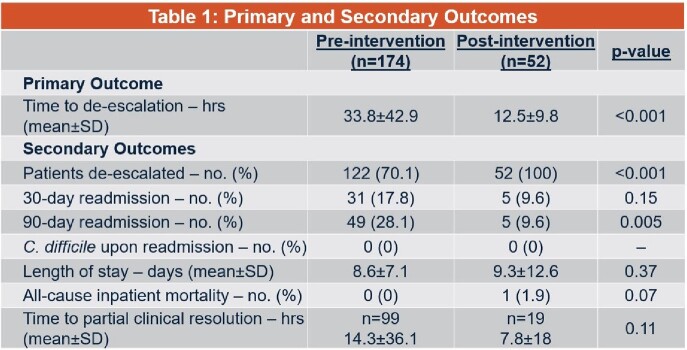

**Conclusion:**

An automatic de-escalation protocol can significantly shorten time to de-escalation in addition to improving rates of de-escalation outside of the ICU without negatively impacting patients.

**Disclosures:**

**All Authors**: No reported disclosures

